# Poly[di-μ-*cis*-cyclo­hexane-1,4-dicarboxyl­ato-μ-*trans*-cyclo­hexane-1,4-dicarboxyl­ato-bis­[dipyrido[3,2-*a*:2′,3′-*c*]phenazine]trimanganese(II)]

**DOI:** 10.1107/S1600536808012737

**Published:** 2008-05-07

**Authors:** Wen-Zhi Zhang, Xiao-Huan Yuan

**Affiliations:** a College of Chemistry and Chemical Engineering, Qiqihar University, Qiqihar 161006, Heilongjiang Province, People’s Republic of China; b Heilongjiang Key Laboratory of Fibrosis Biotherapy, Mudanjiang Medical College, Mudanjiang 157011, Heilongjiang Province, People’s Republic of China

## Abstract

In the title compound, [Mn_3_(C_8_H_10_O_4_)_3_(C_18_H_10_N_4_)_2_], one Mn atom and one cyclohexane-1,4-dicarboxylate (chdc) ligand are located on centres of inversion. One of the two independent Mn atoms is seven-coordinate, binding to five carboxyl­ate O atoms from different chdc ligands and two phenanthrene N atoms from a dipyrido[3,2-*a*:2′,3′-*c*]phenazine (*L*) ligand, while the second Mn atom is six-coordinate, binding to six carboxyl­ate O atoms from different chdc ligands. The *cis*-chdc ligands bridge the trinuclear Mn^II^ clusters, forming chains, which are further linked into a three-dimensional network.

## Related literature

For related structures, see: De (2007 ▶); Li (2007 ▶).
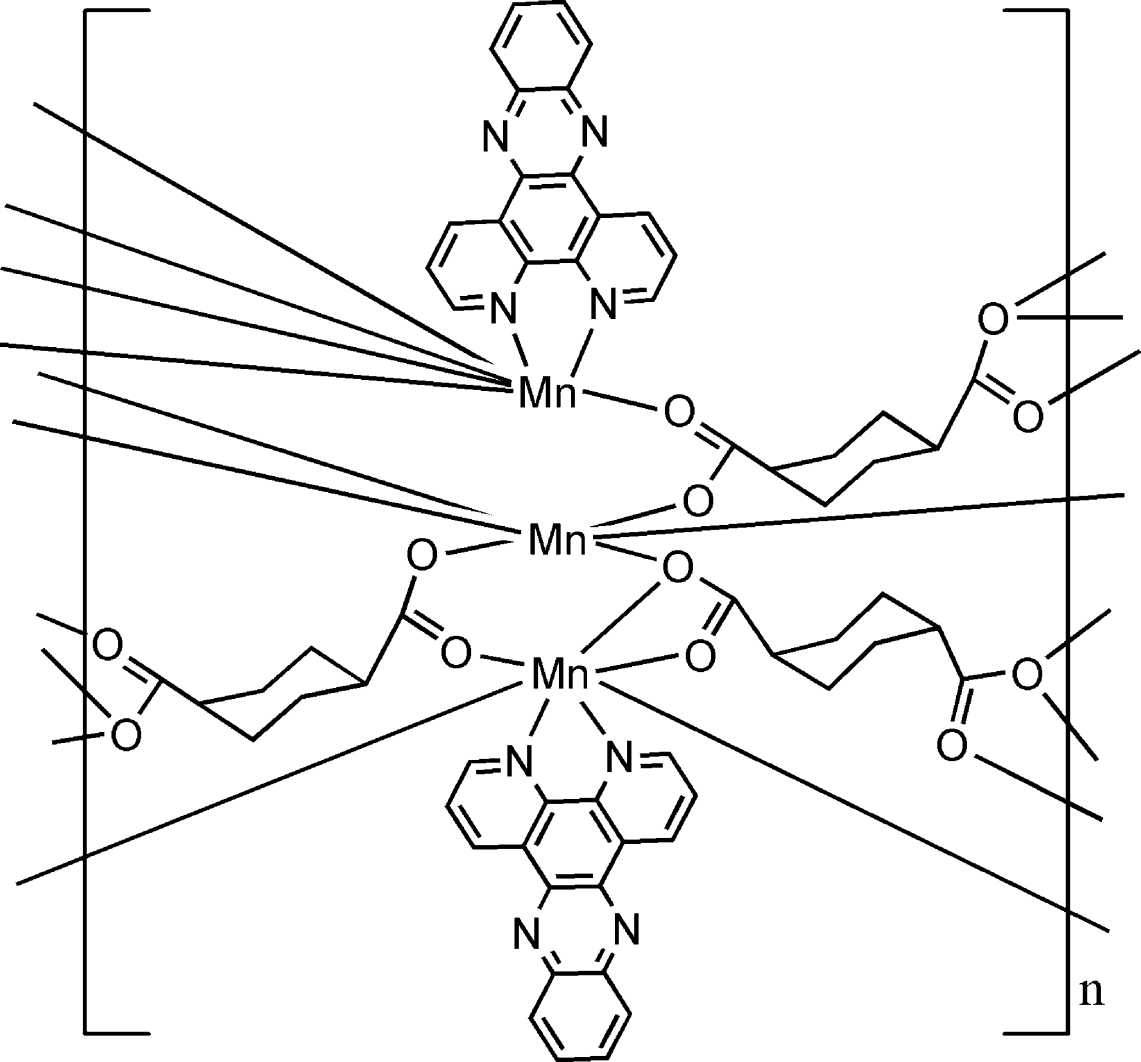


## Experimental

### 

#### Crystal data

[Mn_3_(C_8_H_10_O_4_)_3_(C_18_H_10_N_4_)_2_]
*M* *_r_* = 1239.90
Triclinic, 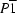

*a* = 8.5730 (17) Å
*b* = 10.614 (2) Å
*c* = 14.846 (3) Å
α = 77.34 (3)°
β = 81.99 (3)°
γ = 82.67 (3)°
*V* = 1298.6 (4) Å^3^
*Z* = 1
Mo *K*α radiation
μ = 0.80 mm^−1^
*T* = 293 (2) K
0.33 × 0.22 × 0.19 mm

#### Data collection

Rigaku R-AXIS RAPID diffractometer
Absorption correction: multi-scan (*ABSCOR*; Higashi, 1995 ▶) *T*_min_ = 0.762, *T*_max_ = 0.863
12776 measured reflections
5830 independent reflections
3707 reflections with *I* > 2σ(*I*)
*R*_int_ = 0.061

#### Refinement

*R*[*F* ^2^ > 2σ(*F* ^2^)] = 0.060
*wR*(*F* ^2^) = 0.176
*S* = 1.05
5830 reflections
376 parameters
H-atom parameters constrained
Δρ_max_ = 0.64 e Å^−3^
Δρ_min_ = −0.74 e Å^−3^

### 

Data collection: *PROCESS-AUTO* (Rigaku, 1998 ▶); cell refinement: *PROCESS-AUTO*; data reduction: *PROCESS-AUTO*; program(s) used to solve structure: *SHELXS97* (Sheldrick, 2008 ▶); program(s) used to refine structure: *SHELXL97* (Sheldrick, 2008 ▶); molecular graphics: *SHELXTL-Plus* (Sheldrick, 2008 ▶); software used to prepare material for publication: *SHELXL97*.

## Supplementary Material

Crystal structure: contains datablocks global, I. DOI: 10.1107/S1600536808012737/bt2704sup1.cif

Structure factors: contains datablocks I. DOI: 10.1107/S1600536808012737/bt2704Isup2.hkl

Additional supplementary materials:  crystallographic information; 3D view; checkCIF report

## Figures and Tables

**Fig. 1.**
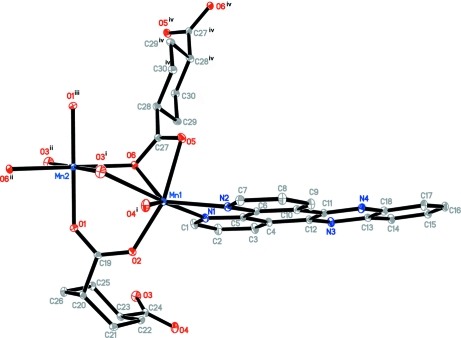
The structure of (I), showing the atomic numbering scheme. Displacement ellipsoids are drawn at the 30% probability level. Symmetry codes: (i) x-1, y, z; (ii) 2-x, 1-y, -z; (iii) 3-x, 1-y, -z; (iv) 2-x, 2-y, -z.

**Fig. 2.**
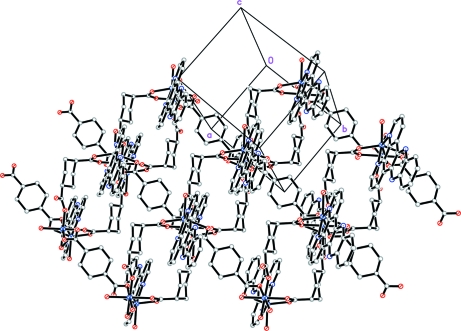
Packing diagram of (I).

## supplementary crystallographic information

### Comment

1,4-Cyclohexanedicarboxylic acid (H_2_chdc), as a flexible multidentate ligand, has been extensively studied in the chemistry of coordination polymers (De, 2007; Li, 2007). Here, we report a new Mn^II^ coordination polymer with chdc ligand, namely [Mn_3_(cis-chdc)_3_(trans-chdc)(*L*)_2_] (I), where *L* = dipyrido[3,2-a:2',3'-c]-phenazine.

In (I) the Mn1 atom is seven-coordinate binding to five carboxylate O atoms from different chdc ligands, and two phenanthrene N atoms from *L* ligand (Fig. 1 and Table 1). The Mn2 atom is six-coordinate binding to six carboxylate O atoms from different chdc ligands (Fig. 1 and Table 1). Interestingly, the chdc ligands bridge neighboring Mn^II^ atoms to give a trinuclear Mn^II^ cluster. The cis-chdc ligands bridge the trinuclear Mn^II^ clusters to form a chain structure, which are further linked   into a 3D network structure (Fig. 2). One Mn atom and one 1,4-cyclohexanedicarboxylate molecule are located on a centre of inversion.

### Experimental

A mixture of Mn(NO_3_)_2_^.^2H_2_O (1 mmol), H_2_chdc (1 mmol) and *L* (1 mmol) was dissolved in 12 ml distilled water, followed by addition of triethylamine until the pH value of the system was approximately 5.5. The resulting solution was sealed in a 23-ml Teflon-lined stainless steel autoclave and heated at 175°C for 8 days under autogenous pressure. The reaction vessel was then slowly cooled to room temperature. Pale yellow block-like crystals of (I) suitable for single-crystal X-ray diffraction analysis were obtained from the resulting solution.

### Refinement

C–bound H atoms were positioned geometrically (C—H = 0.93–0.96 Å) and refined as riding, with *U*_iso_(H) = 1.2*U*_eq_(C).

### Crystal data

**Table:**

[Mn_3_(C_8_H_10_O_4_)_3_(C_18_H_10_N_4_)_2_]	*Z* = 1
*M**_r_* = 1239.90	*F*_000_ = 637
Triclinic, *P*1	*D*_x_ = 1.585 Mg m^−^^3^
Hall symbol: -P 1	Mo *K*α radiation λ = 0.71073 Å
*a* = 8.5730 (17) Å	Cell parameters from 8527 reflections
*b* = 10.614 (2) Å	θ = 3.0–27.5º
*c* = 14.846 (3) Å	µ = 0.80 mm^−^^1^
α = 77.34 (3)º	*T* = 293 (2) K
β = 81.99 (3)º	Block, pale yellow
γ = 82.67 (3)º	0.33 × 0.22 × 0.19 mm
*V* = 1298.6 (4) Å^3^	

### Data collection

**Table:**

Rigaku R-AXIS RAPID diffractometer	5830 independent reflections
Radiation source: rotating anode	3707 reflections with *I* > 2σ(*I*)
Monochromator: graphite	*R*_int_ = 0.062
Detector resolution: 10.0 pixels mm^-1^	θ_max_ = 27.5º
*T* = 293(2) K	θ_min_ = 3.1º
ω scans	*h* = −11→11
Absorption correction: multi-scan(ABSCOR; Higashi, 1995)	*k* = −13→13
*T*_min_ = 0.762, *T*_max_ = 0.863	*l* = −16→19
12776 measured reflections	

### Refinement

**Table:**

Refinement on *F*^2^	Secondary atom site location: difference Fourier map
Least-squares matrix: full	Hydrogen site location: inferred from neighbouring sites
*R*[*F*^2^ > 2σ(*F*^2^)] = 0.060	H-atom parameters constrained
*wR*(*F*^2^) = 0.176	*w* = 1/[σ^2^(*F*_o_^2^) + (0.0908*P*)^2^] where *P* = (*F*_o_^2^ + 2*F*_c_^2^)/3
*S* = 1.05	(Δ/σ)_max_ < 0.001
5830 reflections	Δρ_max_ = 0.64 e Å^−^^3^
376 parameters	Δρ_min_ = −0.74 e Å^−^^3^
Primary atom site location: structure-invariant direct methods	Extinction correction: none

### Special details

**Table:**

Geometry. All esds (except the esd in the dihedral angle between two l.s. planes) are estimated using the full covariance matrix. The cell esds are taken into account individually in the estimation of esds in distances, angles and torsion angles; correlations between esds in cell parameters are only used when they are defined by crystal symmetry. An approximate (isotropic) treatment of cell esds is used for estimating esds involving l.s. planes.
Refinement. Refinement of F^2^ against ALL reflections. The weighted R-factor wR and goodness of fit S are based on F^2^, conventional R-factors R are based on F, with F set to zero for negative F^2^. The threshold expression of F^2^ > 2sigma(F^2^) is used only for calculating R-factors(gt) etc. and is not relevant to the choice of reflections for refinement. R-factors based on F^2^ are statistically about twice as large as those based on F, and R- factors based on ALL data will be even larger.

### Fractional atomic coordinates and isotropic or equivalent isotropic displacement parameters (Å^2^)

**Table:**

	*x*	*y*	*z*	*U*_iso_*/*U*_eq_	
C1	1.3154 (5)	0.6357 (5)	0.2263 (4)	0.0481 (12)	
H1	1.3426	0.6017	0.1729	0.058*	
C2	1.4234 (5)	0.7038 (5)	0.2518 (4)	0.0542 (13)	
H2	1.5216	0.7137	0.2169	0.065*	
C3	1.3834 (5)	0.7570 (5)	0.3297 (4)	0.0513 (13)	
H3	1.4535	0.8044	0.3476	0.062*	
C4	1.2354 (5)	0.7384 (4)	0.3817 (3)	0.0361 (9)	
C5	1.1352 (4)	0.6662 (4)	0.3517 (3)	0.0322 (9)	
C6	0.9812 (5)	0.6398 (4)	0.4043 (3)	0.0312 (8)	
C7	0.7506 (5)	0.5439 (4)	0.4195 (3)	0.0428 (10)	
H7	0.6873	0.4949	0.3977	0.051*	
C8	0.6944 (5)	0.5891 (5)	0.5002 (3)	0.0448 (11)	
H8	0.5957	0.5702	0.5314	0.054*	
C9	0.7855 (5)	0.6617 (4)	0.5334 (3)	0.0405 (10)	
H9	0.7504	0.6915	0.5879	0.049*	
C10	0.9314 (5)	0.6901 (4)	0.4842 (3)	0.0334 (9)	
C11	1.0314 (5)	0.7716 (4)	0.5137 (3)	0.0320 (9)	
C12	1.1837 (5)	0.7951 (4)	0.4643 (3)	0.0361 (9)	
C13	1.2187 (5)	0.9248 (4)	0.5624 (3)	0.0368 (9)	
C14	1.3088 (5)	1.0088 (5)	0.5907 (4)	0.0467 (11)	
H14	1.4085	1.0250	0.5597	0.056*	
C15	1.2492 (5)	1.0660 (4)	0.6635 (3)	0.0455 (11)	
H15	1.3096	1.1204	0.6821	0.055*	
C16	1.0994 (6)	1.0448 (4)	0.7108 (3)	0.0451 (11)	
H16	1.0602	1.0862	0.7596	0.054*	
C17	1.0108 (6)	0.9640 (5)	0.6856 (3)	0.0459 (11)	
H17	0.9116	0.9492	0.7178	0.055*	
C18	1.0681 (5)	0.9021 (4)	0.6110 (3)	0.0372 (9)	
C19	1.2088 (4)	0.3188 (4)	0.1441 (3)	0.0330 (9)	
C20	1.3374 (4)	0.2042 (4)	0.1547 (3)	0.0329 (9)	
H20	1.2888	0.1277	0.1496	0.039*	
C21	1.4043 (5)	0.1721 (4)	0.2488 (3)	0.0371 (9)	
H21A	1.3173	0.1678	0.2983	0.044*	
H21B	1.4648	0.0875	0.2559	0.044*	
C22	1.5100 (4)	0.2725 (4)	0.2577 (3)	0.0329 (9)	
H22A	1.5559	0.2449	0.3158	0.039*	
H22B	1.4464	0.3547	0.2590	0.039*	
C23	1.6420 (4)	0.2913 (4)	0.1774 (3)	0.0344 (9)	
H23	1.7047	0.2070	0.1790	0.041*	
C24	1.7540 (5)	0.3865 (4)	0.1871 (3)	0.0371 (10)	
C25	1.5739 (5)	0.3280 (4)	0.0842 (3)	0.0389 (10)	
H25A	1.5112	0.4115	0.0797	0.047*	
H25B	1.6598	0.3359	0.0340	0.047*	
C26	1.4706 (5)	0.2260 (5)	0.0744 (3)	0.0416 (10)	
H26A	1.5359	0.1447	0.0726	0.050*	
H26B	1.4248	0.2535	0.0161	0.050*	
C27	0.9657 (5)	0.7352 (4)	0.0992 (3)	0.0360 (9)	
C28	0.9835 (5)	0.8607 (4)	0.0286 (3)	0.0426 (10)	
H28	1.0018	0.8391	−0.0332	0.051*	
C29	1.1322 (5)	0.9177 (4)	0.0430 (3)	0.0400 (10)	
H29A	1.1198	0.9368	0.1047	0.048*	
H29B	1.2236	0.8544	0.0383	0.048*	
C30	0.8404 (5)	0.9579 (4)	0.0298 (3)	0.0423 (10)	
H30A	0.8166	0.9787	0.0910	0.051*	
H30B	0.7501	0.9206	0.0172	0.051*	
N1	1.1746 (4)	0.6163 (3)	0.2743 (2)	0.0364 (8)	
N2	0.8915 (4)	0.5678 (3)	0.3718 (2)	0.0340 (8)	
N3	1.2766 (4)	0.8696 (4)	0.4881 (3)	0.0407 (9)	
N4	0.9733 (4)	0.8249 (3)	0.5858 (2)	0.0380 (8)	
O1	1.1606 (4)	0.3541 (3)	0.0659 (2)	0.0532 (9)	
O2	1.1563 (3)	0.3674 (3)	0.2134 (2)	0.0397 (7)	
O3	1.8227 (4)	0.4527 (4)	0.1177 (3)	0.0595 (10)	
O4	1.7807 (4)	0.3935 (3)	0.2666 (2)	0.0532 (9)	
O5	0.8733 (4)	0.7288 (3)	0.1720 (2)	0.0461 (8)	
O6	1.0573 (3)	0.6357 (3)	0.0822 (2)	0.0365 (7)	
Mn1	0.97563 (7)	0.51832 (6)	0.22819 (4)	0.02964 (19)	
Mn2	1.0000	0.5000	0.0000	0.0292 (2)	

### Atomic displacement parameters (Å^2^)

**Table:**

	*U*^11^	*U*^22^	*U*^33^	*U*^12^	*U*^13^	*U*^23^
C1	0.043 (2)	0.049 (3)	0.057 (3)	−0.008 (2)	0.005 (2)	−0.025 (2)
C2	0.038 (2)	0.069 (3)	0.063 (3)	−0.019 (2)	0.008 (2)	−0.031 (3)
C3	0.039 (2)	0.059 (3)	0.066 (3)	−0.016 (2)	0.001 (2)	−0.031 (3)
C4	0.036 (2)	0.034 (2)	0.040 (2)	−0.0096 (17)	0.0023 (18)	−0.0130 (19)
C5	0.033 (2)	0.031 (2)	0.036 (2)	−0.0062 (16)	−0.0048 (17)	−0.0108 (18)
C6	0.038 (2)	0.0253 (19)	0.033 (2)	−0.0071 (16)	−0.0100 (17)	−0.0067 (17)
C7	0.042 (2)	0.041 (2)	0.050 (3)	−0.0163 (19)	−0.006 (2)	−0.013 (2)
C8	0.035 (2)	0.057 (3)	0.047 (3)	−0.017 (2)	0.007 (2)	−0.020 (2)
C9	0.042 (2)	0.044 (3)	0.039 (2)	−0.0143 (19)	0.0044 (19)	−0.015 (2)
C10	0.036 (2)	0.029 (2)	0.038 (2)	−0.0105 (16)	−0.0045 (18)	−0.0087 (18)
C11	0.038 (2)	0.029 (2)	0.033 (2)	−0.0069 (16)	−0.0070 (17)	−0.0105 (17)
C12	0.036 (2)	0.035 (2)	0.042 (2)	−0.0061 (17)	−0.0077 (18)	−0.015 (2)
C13	0.038 (2)	0.032 (2)	0.044 (3)	−0.0054 (17)	−0.0104 (19)	−0.0127 (19)
C14	0.037 (2)	0.054 (3)	0.058 (3)	−0.010 (2)	−0.005 (2)	−0.028 (2)
C15	0.047 (3)	0.042 (3)	0.057 (3)	−0.008 (2)	−0.013 (2)	−0.023 (2)
C16	0.057 (3)	0.043 (3)	0.042 (3)	−0.007 (2)	−0.007 (2)	−0.020 (2)
C17	0.052 (3)	0.051 (3)	0.040 (3)	−0.019 (2)	0.004 (2)	−0.020 (2)
C18	0.041 (2)	0.034 (2)	0.040 (2)	−0.0120 (18)	−0.0062 (19)	−0.0084 (19)
C19	0.033 (2)	0.0235 (19)	0.044 (2)	−0.0064 (16)	−0.0099 (18)	−0.0058 (18)
C20	0.031 (2)	0.0238 (19)	0.047 (2)	−0.0073 (15)	−0.0061 (18)	−0.0107 (18)
C21	0.036 (2)	0.030 (2)	0.044 (3)	−0.0092 (17)	−0.0082 (18)	0.0013 (19)
C22	0.0299 (19)	0.037 (2)	0.034 (2)	−0.0113 (16)	−0.0075 (16)	−0.0051 (18)
C23	0.0288 (19)	0.030 (2)	0.047 (3)	−0.0068 (16)	−0.0013 (18)	−0.0136 (19)
C24	0.031 (2)	0.037 (2)	0.047 (3)	−0.0098 (17)	0.0017 (19)	−0.017 (2)
C25	0.038 (2)	0.044 (2)	0.037 (2)	−0.0093 (18)	−0.0001 (18)	−0.013 (2)
C26	0.035 (2)	0.052 (3)	0.044 (3)	−0.0067 (19)	−0.0022 (19)	−0.022 (2)
C27	0.047 (2)	0.028 (2)	0.037 (2)	−0.0106 (18)	−0.009 (2)	−0.0073 (18)
C28	0.053 (3)	0.026 (2)	0.048 (3)	−0.0078 (18)	−0.001 (2)	−0.0045 (19)
C29	0.039 (2)	0.031 (2)	0.047 (3)	−0.0050 (18)	−0.003 (2)	−0.003 (2)
C30	0.045 (2)	0.032 (2)	0.050 (3)	−0.0124 (18)	−0.005 (2)	−0.004 (2)
N1	0.0386 (18)	0.0342 (19)	0.039 (2)	−0.0080 (15)	−0.0005 (15)	−0.0128 (16)
N2	0.0337 (17)	0.0320 (18)	0.040 (2)	−0.0101 (14)	−0.0060 (15)	−0.0099 (16)
N3	0.0353 (18)	0.045 (2)	0.049 (2)	−0.0119 (15)	−0.0009 (16)	−0.0233 (19)
N4	0.0442 (19)	0.041 (2)	0.0338 (19)	−0.0150 (16)	−0.0048 (16)	−0.0112 (16)
O1	0.069 (2)	0.0470 (19)	0.048 (2)	0.0177 (16)	−0.0304 (17)	−0.0172 (16)
O2	0.0408 (16)	0.0360 (16)	0.0435 (18)	0.0027 (12)	−0.0073 (14)	−0.0129 (14)
O3	0.055 (2)	0.063 (2)	0.063 (2)	−0.0343 (18)	0.0056 (17)	−0.0086 (19)
O4	0.0518 (19)	0.061 (2)	0.059 (2)	−0.0265 (16)	−0.0042 (16)	−0.0263 (18)
O5	0.0587 (19)	0.0331 (16)	0.0444 (19)	−0.0054 (14)	0.0034 (16)	−0.0088 (14)
O6	0.0482 (16)	0.0239 (14)	0.0425 (17)	−0.0089 (12)	−0.0098 (13)	−0.0115 (13)
Mn1	0.0326 (3)	0.0273 (3)	0.0316 (4)	−0.0081 (2)	−0.0036 (3)	−0.0088 (3)
Mn2	0.0341 (4)	0.0253 (4)	0.0306 (5)	−0.0070 (3)	−0.0073 (4)	−0.0067 (4)

### Geometric parameters (Å, °)

**Table:**

C1—N1	1.329 (5)	C21—H21A	0.9700
C1—C2	1.383 (6)	C21—H21B	0.9700
C1—H1	0.9300	C22—C23	1.523 (6)
C2—C3	1.379 (6)	C22—H22A	0.9700
C2—H2	0.9300	C22—H22B	0.9700
C3—C4	1.403 (6)	C23—C24	1.520 (5)
C3—H3	0.9300	C23—C25	1.529 (6)
C4—C5	1.396 (5)	C23—H23	0.9800
C4—C12	1.472 (5)	C24—O3	1.234 (5)
C5—N1	1.351 (5)	C24—O4	1.253 (5)
C5—C6	1.464 (5)	C25—C26	1.526 (6)
C6—N2	1.351 (5)	C25—H25A	0.9700
C6—C10	1.397 (5)	C25—H25B	0.9700
C7—N2	1.337 (5)	C26—H26A	0.9700
C7—C8	1.389 (6)	C26—H26B	0.9700
C7—H7	0.9300	C27—O5	1.243 (5)
C8—C9	1.368 (6)	C27—O6	1.283 (5)
C8—H8	0.9300	C27—C28	1.514 (6)
C9—C10	1.391 (6)	C28—C30	1.500 (6)
C9—H9	0.9300	C28—C29	1.539 (6)
C10—C11	1.461 (5)	C28—H28	0.9800
C11—N4	1.325 (5)	C29—C30^i^	1.533 (6)
C11—C12	1.428 (6)	C29—H29A	0.9700
C12—N3	1.322 (5)	C29—H29B	0.9700
C13—N3	1.365 (5)	C30—C29^i^	1.533 (6)
C13—C18	1.411 (6)	C30—H30A	0.9700
C13—C14	1.414 (6)	C30—H30B	0.9700
C14—C15	1.360 (6)	N1—Mn1	2.356 (3)
C14—H14	0.9300	N2—Mn1	2.303 (3)
C15—C16	1.395 (7)	O1—Mn2	2.102 (3)
C15—H15	0.9300	O2—Mn1	2.107 (3)
C16—C17	1.356 (6)	O3—Mn2^ii^	2.165 (3)
C16—H16	0.9300	O3—Mn1^ii^	2.495 (4)
C17—C18	1.408 (6)	O4—Mn1^ii^	2.200 (3)
C17—H17	0.9300	O5—Mn1	2.312 (3)
C18—N4	1.364 (5)	O6—Mn2	2.218 (3)
C19—O1	1.251 (5)	O6—Mn1	2.314 (3)
C19—O2	1.253 (5)	Mn1—O4^iii^	2.200 (3)
C19—C20	1.531 (5)	Mn1—O3^iii^	2.495 (4)
C20—C26	1.531 (6)	Mn2—O1^iv^	2.102 (3)
C20—C21	1.536 (6)	Mn2—O3^v^	2.165 (3)
C20—H20	0.9800	Mn2—O3^iii^	2.165 (3)
C21—C22	1.521 (5)	Mn2—O6^iv^	2.218 (3)
			
N1—C1—C2	123.2 (4)	C23—C25—H25A	109.5
N1—C1—H1	118.4	C26—C25—H25B	109.5
C2—C1—H1	118.4	C23—C25—H25B	109.5
C3—C2—C1	119.0 (4)	H25A—C25—H25B	108.0
C3—C2—H2	120.5	C25—C26—C20	111.9 (3)
C1—C2—H2	120.5	C25—C26—H26A	109.2
C2—C3—C4	119.0 (4)	C20—C26—H26A	109.2
C2—C3—H3	120.5	C25—C26—H26B	109.2
C4—C3—H3	120.5	C20—C26—H26B	109.2
C5—C4—C3	118.0 (4)	H26A—C26—H26B	107.9
C5—C4—C12	120.2 (3)	O5—C27—O6	121.1 (4)
C3—C4—C12	121.8 (4)	O5—C27—C28	122.5 (4)
N1—C5—C4	122.5 (4)	O6—C27—C28	116.3 (4)
N1—C5—C6	116.7 (3)	C30—C28—C27	114.1 (4)
C4—C5—C6	120.8 (3)	C30—C28—C29	111.5 (3)
N2—C6—C10	122.6 (4)	C27—C28—C29	108.7 (4)
N2—C6—C5	117.5 (3)	C30—C28—H28	107.4
C10—C6—C5	119.9 (3)	C27—C28—H28	107.4
N2—C7—C8	122.9 (4)	C29—C28—H28	107.4
N2—C7—H7	118.5	C30^i^—C29—C28	110.7 (4)
C8—C7—H7	118.5	C30^i^—C29—H29A	109.5
C9—C8—C7	119.5 (4)	C28—C29—H29A	109.5
C9—C8—H8	120.3	C30^i^—C29—H29B	109.5
C7—C8—H8	120.3	C28—C29—H29B	109.5
C8—C9—C10	118.8 (4)	H29A—C29—H29B	108.1
C8—C9—H9	120.6	C28—C30—C29^i^	111.0 (4)
C10—C9—H9	120.6	C28—C30—H30A	109.4
C9—C10—C6	118.5 (4)	C29^i^—C30—H30A	109.4
C9—C10—C11	121.6 (4)	C28—C30—H30B	109.4
C6—C10—C11	119.9 (3)	C29^i^—C30—H30B	109.4
N4—C11—C12	121.9 (4)	H30A—C30—H30B	108.0
N4—C11—C10	117.4 (4)	C1—N1—C5	118.2 (4)
C12—C11—C10	120.6 (3)	C1—N1—Mn1	125.1 (3)
N3—C12—C11	122.4 (3)	C5—N1—Mn1	116.4 (2)
N3—C12—C4	119.1 (4)	C7—N2—C6	117.6 (3)
C11—C12—C4	118.5 (3)	C7—N2—Mn1	124.1 (3)
N3—C13—C18	121.2 (4)	C6—N2—Mn1	117.8 (3)
N3—C13—C14	120.0 (4)	C12—N3—C13	116.4 (3)
C18—C13—C14	118.8 (4)	C11—N4—C18	116.3 (3)
C15—C14—C13	119.8 (4)	C19—O1—Mn2	137.6 (3)
C15—C14—H14	120.1	C19—O2—Mn1	130.2 (3)
C13—C14—H14	120.1	C24—O3—Mn2^ii^	158.1 (3)
C14—C15—C16	121.5 (4)	C24—O3—Mn1^ii^	86.0 (3)
C14—C15—H15	119.3	Mn2^ii^—O3—Mn1^ii^	93.87 (12)
C16—C15—H15	119.3	C24—O4—Mn1^ii^	99.5 (3)
C17—C16—C15	120.0 (4)	C27—O5—Mn1	91.5 (2)
C17—C16—H16	120.0	C27—O6—Mn2	124.5 (3)
C15—C16—H16	120.0	C27—O6—Mn1	90.3 (2)
C16—C17—C18	120.5 (4)	Mn2—O6—Mn1	97.62 (11)
C16—C17—H17	119.8	O2—Mn1—O4^iii^	95.86 (12)
C18—C17—H17	119.8	O2—Mn1—N2	120.52 (12)
N4—C18—C17	118.7 (4)	O4^iii^—Mn1—N2	82.87 (12)
N4—C18—C13	121.8 (4)	O2—Mn1—O5	147.38 (12)
C17—C18—C13	119.5 (4)	O4^iii^—Mn1—O5	108.96 (13)
O1—C19—O2	124.9 (4)	N2—Mn1—O5	84.24 (12)
O1—C19—C20	116.1 (3)	O2—Mn1—O6	91.43 (11)
O2—C19—C20	118.9 (4)	O4^iii^—Mn1—O6	127.44 (12)
C19—C20—C26	110.3 (4)	N2—Mn1—O6	135.18 (11)
C19—C20—C21	114.4 (3)	O5—Mn1—O6	56.78 (10)
C26—C20—C21	110.8 (3)	O2—Mn1—N1	84.36 (11)
C19—C20—H20	107.0	O4^iii^—Mn1—N1	148.93 (13)
C26—C20—H20	107.0	N2—Mn1—N1	70.66 (12)
C21—C20—H20	107.0	O5—Mn1—N1	84.83 (12)
C22—C21—C20	112.2 (3)	O6—Mn1—N1	83.54 (12)
C22—C21—H21A	109.2	O2—Mn1—O3^iii^	91.42 (12)
C20—C21—H21A	109.2	O4^iii^—Mn1—O3^iii^	54.17 (12)
C22—C21—H21B	109.2	N2—Mn1—O3^iii^	129.79 (12)
C20—C21—H21B	109.2	O5—Mn1—O3^iii^	86.59 (12)
H21A—C21—H21B	107.9	O6—Mn1—O3^iii^	73.72 (11)
C21—C22—C23	111.7 (3)	N1—Mn1—O3^iii^	156.77 (12)
C21—C22—H22A	109.3	O1^iv^—Mn2—O1	180.00 (17)
C23—C22—H22A	109.3	O1^iv^—Mn2—O3^v^	89.57 (15)
C21—C22—H22B	109.3	O1—Mn2—O3^v^	90.43 (15)
C23—C22—H22B	109.3	O1^iv^—Mn2—O3^iii^	90.43 (15)
H22A—C22—H22B	107.9	O1—Mn2—O3^iii^	89.57 (15)
C24—C23—C22	112.7 (3)	O3^v^—Mn2—O3^iii^	180.0 (2)
C24—C23—C25	112.4 (4)	O1^iv^—Mn2—O6	89.89 (11)
C22—C23—C25	110.7 (3)	O1—Mn2—O6	90.11 (11)
C24—C23—H23	106.9	O3^v^—Mn2—O6	97.56 (12)
C22—C23—H23	106.9	O3^iii^—Mn2—O6	82.44 (12)
C25—C23—H23	106.9	O1^iv^—Mn2—O6^iv^	90.11 (11)
O3—C24—O4	120.0 (4)	O1—Mn2—O6^iv^	89.89 (11)
O3—C24—C23	120.7 (4)	O3^v^—Mn2—O6^iv^	82.44 (12)
O4—C24—C23	119.2 (4)	O3^iii^—Mn2—O6^iv^	97.56 (12)
C26—C25—C23	110.9 (4)	O6—Mn2—O6^iv^	180.00 (11)
C26—C25—H25A	109.5		

Symmetry codes: (i) −*x*+2, −*y*+2, −*z*; (ii) *x*+1, *y*, *z*; (iii) *x*−1, *y*, *z*; (iv) −*x*+2, −*y*+1, −*z*; (v) −*x*+3, −*y*+1, −*z*.
